# Compensatory Motor Neuron Response to Chromatolysis in the Murine hSOD1^G93A^ Model of Amyotrophic Lateral Sclerosis

**DOI:** 10.3389/fncel.2014.00346

**Published:** 2014-10-22

**Authors:** Javier Riancho, Maria Ruiz-Soto, Nuria T. Villagrá, Jose Berciano, Maria T. Berciano, Miguel Lafarga

**Affiliations:** ^1^Service of Neurology, University Hospital Marqués de Valdecilla, Instituto de Investigación Valdecilla (IDIVAL), University of Cantabria, Santander, Spain; ^2^Department of Anatomy and Cell Biology, Centro de Investigación Biomédica en Red sobre Enfermedades Neurodegenerativas (CIBERNED), Instituto de Investigación Valdecilla, University of Cantabria, Santander, Spain; ^3^Service of Pathology, University Hospital Marqués de Valdecilla, Instituto de Investigación Valdecilla, University of Cantabria, Santander, Spain

**Keywords:** neurodegeneration, amyotrophic lateral sclerosis, chromatolysis, stress granules, perinuclear region, nucleolus, rRNA transcription, endoplasmic reticulum stress

## Abstract

We investigated neuronal self-defense mechanisms in a murine model of amyotrophic lateral sclerosis (ALS), the transgenic hSOD1^G93A^, during both the asymptomatic and symptomatic stages. This is an experimental model of endoplasmic reticulum (ER) stress with severe chromatolysis. As a compensatory response to translation inhibition, chromatolytic neurons tended to reorganize the protein synthesis machinery at the perinuclear region, preferentially at nuclear infolding domains enriched in nuclear pores. This organization could facilitate nucleo-cytoplasmic traffic of RNAs and proteins at translation sites. By electron microscopy analysis, we observed that the active euchromatin pattern and the reticulated nucleolar configuration of control motor neurons were preserved in ALS chromatolytic neurons. Moreover the 5′-fluorouridine (5′-FU) transcription assay, at the ultrastructural level, revealed high incorporation of the RNA precursor 5′-FU into nascent RNA. Immunogold particles of 5′-FU incorporation were distributed throughout the euchromatin and on the dense fibrillar component of the nucleolus in both control and ALS motor neurons. The high rate of rRNA transcription in ALS motor neurons could maintain ribosome biogenesis under conditions of severe dysfunction of proteostasis. Collectively, the perinuclear reorganization of protein synthesis machinery, the predominant euchromatin architecture, and the active nucleolar transcription could represent compensatory mechanisms in ALS motor neurons in response to the disturbance of ER proteostasis. In this scenario, epigenetic activation of chromatin and nucleolar transcription could have important therapeutic implications for neuroprotection in ALS and other neurodegenerative diseases. Although histone deacetylase inhibitors are currently used as therapeutic agents, we raise the untapped potential of the nucleolar transcription of ribosomal genes as an exciting new target for the therapy of some neurodegenerative diseases.

## Introduction

Amyotrophic lateral sclerosis (ALS) is an adult-onset neurodegenerative disease characterized by degeneration of motor neurons in the anterior spinal horn, brainstem nuclei, and cerebral cortex (Rowland and Shneider, 2001). An important component in ALS cellular pathophysiology is a chronic stress response at the endoplasmic reticulum (ER) leading to severe disruption of proteostasis. An essential function of the rough ER (RER) is the synthesis of secretory, membrane, and lysosomal proteins, as well as their folding and quality control. Different cellular stressors cause protein misfolding at the RER with inhibition of protein translation, a condition called “ER stress” (Hetz, 2012). As a compensatory mechanism against ER stress, neurons may activate the unfolded protein response (UPR) that, depending on the intensity and nature of the stress stimuli, may lead to either the correct protein folding (neuroprotection) or to neurodegeneration (Hetz and Mollereau, 2014). Neuronal manifestations of chronic ER stress are fragmentation and dissolution of the RER cisterns, an alteration referred to as chromatolysis, and accumulation of misfolded and aberrant proteins in cytoplasmic inclusions. These two cellular events commonly occur in motor neurons of both ALS patients and murine experimental models of the disease (Kusaka et al., 1988; Martin, 1999; Oyanagi et al., 2008; Sasaki, 2010).

Chromatolysis is a prominent neuropathological feature induced by axonal injury, ischemia, neurotoxicity, and several neurodegenerative disorders such as ALS, spinal muscular atrophy, and Alzheimer’s disease (Barr and Bertram, 1951; Lieberman, 1971; Wakayama, 1992; Sasaki, 2011; Tapia et al., 2012; Palanca et al., 2014a). Chromatolytic dissolution of the RER clearly reflects a dysfunction of protein synthesis that most often precedes apoptosis (Martin, 1999). However, neuronal recovery can occur following axotomy and treatment with certain neurotoxic agents (Lieberman, 1971; Palanca et al., 2014a) due to successful activation of neuroprotective mechanisms for neuronal survival.

A major component to maintain the structural and functional integrity of the RER is ribosome biogenesis, which is dynamically accommodated to the cellular demands for protein synthesis. The nucleolus is the nuclear factory for rRNA synthesis, maturing rRNA transcripts, and preribosome subunit assembly (Raska et al., 2006; Boisvert et al., 2007; Grummt, 2013). Beyond its role in ribosome biogenesis, the nucleolus constitutes a central hub for sensing and coordinating cellular stress responses (Olson, 2004; Boulon et al., 2010; Kreiner et al., 2013; Parlato and Liss, 2014). Nucleoli are very prominent in mammalian projection neurons to sustain the high rate of ribosome biogenesis required for protein synthesis. Neurons can accommodate changes in the demand for protein synthesis by regulating the number and size of nucleoli, as well as the transcriptional activity of ribosomal genes (Lafarga et al., 1991; Berciano et al., 2007; Jordan et al., 2007; Hetman and Pietrzak, 2012; Palanca et al., 2014b). In fact, nucleolar dysfunction has been involved in the pathophysiology of several neurodegenerative diseases (Hetman et al., 2010; Baltanas et al., 2011a; Hetman and Pietrzak, 2012; Parlato and Kreiner, 2013; Lee et al., 2014).

An important question in motor neuron diseases is how chromatolytic neurons can survive under conditions of severe disturbance of protein synthesis and ER proteostasis, before the final activation of the apoptotic program. In an experimental model of reversible chromatolysis induced by proteasome inhibition in rat sensory ganglion neurons, we have recently demonstrated that the reorganization of the nuclear envelope environment and the hyperactivity of the nucleolus play a key neuroprotective role in maintaining neuronal survival (Palanca et al., 2014b). In the present work we used an animal model of ALS, the transgenic hSOD1^G93A^ mouse (Gurney et al., 1994; Sunico et al., 2011; Mòdol et al., 2014), to investigate the cellular basis of both chromatolysis and compensatory neuroprotective mechanisms in affected spinal motor neurons. We demonstrate that the dysfunction of the RER is associated with the sequestration of RNA and the preinitiation factor of translation eIF3 in stress granules (SGs). Moreover, to mitigate chromatolysis-mediated inhibition of translation, motor neurons reorganize the protein synthesis machinery at the perinuclear region, preserve the transcriptionally active euchromatin domains, and maintain an active nucleolar transcription for ribosome biogenesis.

## Materials and Methods

### Animals

Transgenic mice [B6SJLTg (SOD1-G93A) 1Gur/J] were obtained from The Jackson Laboratory (Bar Harbor, ME, USA) and maintained at the Animal Service of the University of Cantabria. The colony was maintained by mating heterozygous transgenic males with B6SJLF1/J hybrid females. Real time quantitative PCR of DNA obtained from tail tissue was used for genotyping, with specific primers detecting human SOD1 and the housekeeping mouse gene ApoB. Primer sequences were: SOD1: GGG AAG CTG TTG TCC CAA G and CAA GGG GAG GTA AAA GAG AGC; ApoB: TCA CCA GTC ATT TCT GCC TTT G and GGG AAG CTG TTG TCC CAA G. Transgenic mice and control littermates were housed under controlled temperature and humidity, with a 12-h light/dark cycle and free access to water and food. Animal care and handling was in accordance with Spanish legislation (Spanish Royal Decree 53/2013 BOE) and the guidelines of the European Commission for the accommodation and care of laboratory animals (revised in Appendix A of the Council Directive 2010/63/UE). The experimental plan was preliminarily examined and approved by the Ethics Committee of the University of Cantabria. Animal sacrifice was performed under deep pentobarbital anesthesia (50 mg/kg). In order to evaluate both presymptomatic and symptomatic stage, transgenic and control mice were sacrificed at day 65 and 105 of life.

### Case report

A 53 years-old previously healthy man was admitted to our Hospital referring to a progressive history of gait difficulties during last 3 years. The neurological exam showed a generalized pyramidal syndrome. Once all the complementary exams were performed, a suspicious diagnosis of primary lateral sclerosis was done. Nevertheless 1 year after diagnosis, symptoms and signs of lower motor neuron degeneration were evidenced, being finally diagnosed of ALS. The patient continued deteriorating and died 5 years after the clinical onset. As control subject, a 67-year-old man with no evidence of neurological disorder was used. Post-mortem examination of spinal cord tissue samples was conducted after written consent given by a near kin. In both cases, autopsy was performed 8 h after death.

### Light microscopy and immunofluorescence

For light microscopy analysis, control and transgenic hSOD1^G93A^ mice were perfused under deep anesthesia with pentobarbital (50 mg/kg) with 3.7% paraformaldehyde (freshly prepared) in PBS. After fixation, the lumbar segment of the spinal cord was removed and washed in PBS. Tissue fragments from the anterior horn were transferred to a drop of PBS on a siliconized slide and squash preparations of dissociated neurons were performed following the procedure previously reported (Pena et al., 2001). Human tissue samples from the lumbar segment were fixed with 3.7% paraformaldehyde. Anterior horn tissue fragments were dissected out from 300 μm thick vibratome sections and processed for neuronal dissociation as indicated above.

For immunofluorescence, the samples were, then, sequentially treated with 0.5% Triton X-100 in PBS for 45 min, 0.1 M glycine in PBS containing 1% bovine serum albumin (BSA) for 30 min and 0.05% Tween 20 in PBS for 5 min. The samples were incubated for 3 h with the primary antibody, goat anti-eIF3η (Santa Cruz Lab, sc-16377, 1:200, La Jolla, CA, USA), containing 1% BSA at room temperature, washed with 0.05% Tween 20 in PBS, incubated for 45 min in the specific secondary antibody conjugated with FITC (Jackson, USA), washed in PBS, counterstained with propidium iodide, and mounted with the ProLong anti-fading medium (Invitrogen). Confocal images were obtained with a LSM510 (Zeiss, Germany) laser scanning microscope and using a 63 × oil (1.4 NA) objective. In order to avoid overlapping signals, images were obtained by sequential excitation at 488 and 543 nm. Images were processed using Photoshop software.

The morphometric analysis of the nucleolar size was performed in dissociated motor neurons with diameter larger than 20 μm. Neuronal preparations were stained with propidium iodide, the nucleolar diameter in mononucleolated neurons being measured on confocal images using a 63 × oil objective and the LSM510 software for morphometric analysis. At least 100 neurons for each experimental group (control, presymptomatic ALS mice, and symptomatic ALS mice) were analyzed. Data were analyzed using one-way ANOVA followed by Bonferroni tests for comparisons. Statistical significance was set at *p* < 0.05. All the analyses were carried out using GraphPad software for Windows.

### Electron microscopy

For conventional ultrastructural examination of motor neurons, control and transgenic hSOD1^G93A^ mice (*n* = 3 per group) were perfused under deep anesthesia with 1% paraformaldehyde and 1% glutaraldehyde in 0.1 M phosphate buffer, pH 7.4. The lumbar segment of the spinal cord was removed. The anterior horn was dissected from 300 μm thick Vibratome sections. Human tissue fragments from the lumbar segment of the anterior horn were fixed with 3% glutaraldehyde in 0.1 M phosphate buffer. Mice and human tissue fragments were rinsed in 0.1 M phosphate buffer, post-fixed in 2% osmium tetroxide, dehydrated in acetone, and embedded in araldite (Durcupan, Fluka, Switzerland). Semithin sections (1 μm thick) stained with toluidine blue were used for light microscopy studies and for the quantitative analysis of the frequency of motor neurons having perinuclear caps of RER. Three animal per animal group (wild type, and asymptomatic and symptomatic ALS mice) and 100 neurons per animal were used. Ultrathin sections stained with uranyl acetate and lead citrate were examined with a JEOL 201 electron microscope.

### Run on transcription assay with 5′-fluorouridine

Active transcription sites were labeled by the incorporation of 5′-fluorouridine (5′-FU) into nascent RNA, as previously reported (Casafont et al., 2006). Briefly, under anesthesia both control and transgenic hSOD1^G93A^ mice (*n* = 3 per group) were given an intravenous injection of 5′-FU (Sigma, UK) at doses of 5 μl/g of a stock solution of 0.4 M 5′-FU in 0.9% saline. All animals were sacrificed after 45 min post-injection of the halogenated nucleotide and fixed by perfusion with 3.7% paraformaldehyde in 0.1 M cacodylate buffer for 15 min at room temperature. Small tissue fragments of the anterior horn were washed in 0.1 M cacodylate buffer, dehydrated in increasing concentrations of methanol at -20°C, embedded in Lowicryl K4 M at -20°C and polymerized with ultraviolet irradiation. Ultrathin sections (60 nm thick) were mounted on nickel grids and sequentially incubated with 0.1 M glycine in PBS for 15 min, 5% BSA in PBS for 30 min, and the primary mouse monoclonal anti-BrdU antibody (clone BU-33, Sigma-Aldrich, UK), diluted 1:25 in 50 mM Tris–HCl, pH 7.6, containing 1% BSA and 0.1 M glycine, for 1 h at 37°C. After washing, the sections were incubated with the specific secondary antibodies coupled to 15-nm gold particles (BioCell, UK; diluted 1:50 in PBS containing 1% BSA). Following immunogold labeling, the grids were stained with lead citrate and uranyl acetate and examined with a JEOL 201 electron microscope. As controls, ultrathin sections were treated as described above but without using the primary antibody.

The quantitative analysis of labeling density was performed on electron microscopy images, recorded at a magnification of 25,000×, of nucleus and nucleoli of motor neurons from three wild type and three symptomatic transgenic hSOD1^G93A^ mice. Labeling density values of gold particles lying on euchromatin and nucleoli, expressed as number of particles per 1 μm^2^, were determined using the ImageJ software (US National Institutes of Health, Bethesda, MD, USA). Electron micrographs from 15 motor neurons per animal were sampled.

## Results

### Stress granules are induced in motor neurons of both affected transgenic hSOD1^G93A^ mice and the ALS patient

**Figure 1:**
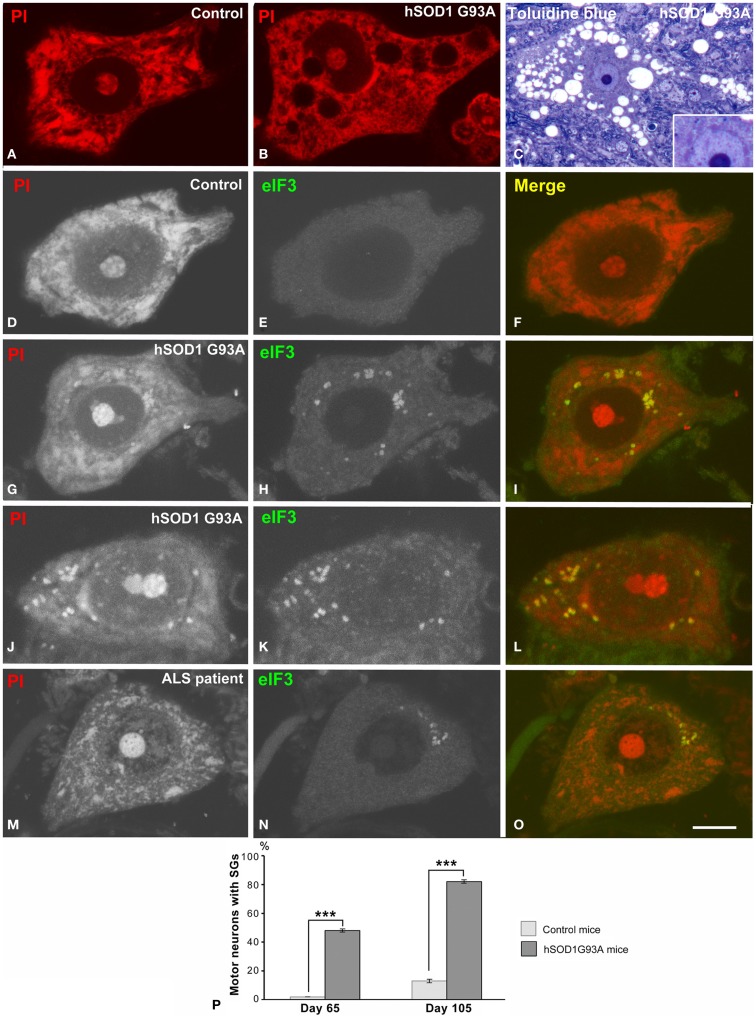
**(A,B)** Dissociated spinal motor neurons stained with propidium iodide (PI) from control **(A)** and hSOD1^G93A^ mice **(B)**. Note the prominent Nissl bodies and nucleolus in the control neuron **(A)**. The hSOD1^G93A^ motor neuron exhibits dispersion of the Nissl substance, excluding a perinuclear cap strongly stained with PI, and vacuolar cytoplasmic degeneration. **(C)** Toluidine blue staining of an ALS motor neuron illustrating the extensive vacuolar degeneration. Note the prominent nucleolus, the pale euchromatic nucleus, and the basophilic perinuclear cap (inset). **(D–L)** Dissociated motor neurons from control **(D–F)** and hSOD1^G93A^ mice immunolabeled for eIF3 and costained with PI. eIF3 shows a diffuse cytoplasmic staining in the control neuron **(E)**, but it is concentrated in SGs in ALS motor neurons **(H,K)**. Note the colocalization of eIF3 and RNA (stained with PI) in SGs. **(M–O)** Representative example of a motor neuron from the ALS patient illustrating the presence of eIF3-positive granules. Scale bars: **(A,B,D–L)** = 7 μm; **(C)** = 18 μm, inset 10 μm; **(M–O)** = 8.5 μm. **(P)** Quantitative analysis of the percentage of motor neurons from control and hSOD1^G93A^ mice with eIF3-positive SGs. Data are mean ± SE from three independent experiments, ***p < 0.001.

Spinal motor neurons in wild type (controls) and transgenic hSOD1^G93A^ mice were examined in preparations of dissociated neurons (Pena et al., 2001) and 1 μm semithin sections. The cytochemical staining for nucleic acids with propidium iodide revealed prominent rRNA-rich nucleoli and Nissl bodies in control neurons. At the symptomatic stage (105 days old), a disintegration of Nissl bodies associated with vacuolar degeneration of perikaryal cytoplasm and neuronal processes were cytological hallmarks in hSOD1^G93A^ neurons, although the prominent nucleoli were preserved (Figures 1A–C).

Since disruption of the protein synthesis machinery (Nissl bodies) with accumulation of misfolded proteins is a manifestation of the ER stress response in the ALS-linked SOD1 mutants (Kikuchi et al., 2006), we analyzed whether this mutation induced the formation of SGs containing stellated translational initiation complexes (Kedersha et al., 2013). Co-staining for nucleic acids and the immunocytochemical marker of SGs eIF3η, the largest initiation factor of translation (Malys and McCarthy, 2011), revealed a diffuse cytoplasmic eIF3η immunostaining in control neurons and the concentration of this factor in numerous SGs in transgenic hSOD1 neurons. The presence of eIF3η-positive SGs was also confirmed in motor neurons of the ALS patient (Figures 1M–O). Sudan black was used to remove endogenous autofluorescence due to lipofuscin in both mouse and human neuronal samples (Liu-Yesucevitz et al., 2010). Interestingly, SGs also sequestrated RNAs not associated with polyribosomes as a result of the translational inhibition. The quantitative analysis of the proportion of motor neurons containing SGs revealed a significant increase from the asymptomatic (65 days old) to symptomatic (105 days old) stages in ALS mice, while they were rarely found in control neurons (Figure 1P).

### Chromatolytic disruption of the RER in hSOD1^G93A^ motor neurons

**Figure 2:**
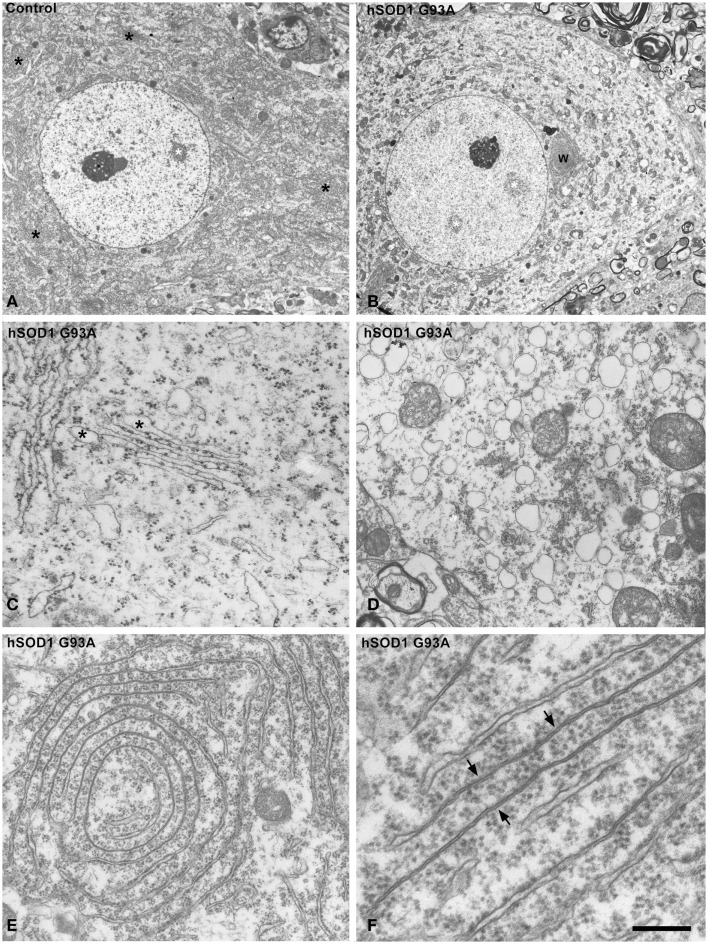
**(A,B)** Electron micrographs of motor neurons from control **(A)** and hSOD1^G93A^ mice **(B)**. While control neuron exhibits numerous Nissl bodies throughout the cytoplasm (black asterisks), the ALS neuron shows a pale extensive chromatolytic area free of Nissl bodies. Note, however, that both neurons display large euchromatic nuclei with several interchromatin granule clusters (white asterisks) and a prominent nucleolus. W: whorl of RER. **(C,D)** Disruption of the protein synthesis machinery in ALS motor neurons from the hSOD1^G93A^ mice. Note in **(C)**, the partial detachment of polyribosomes from RER cisterns, with numerous polyribosomes scattered throughout the cytosol, and the focal dilations of cisterns (asterisks). **(D)** illustrates a cytoplasmic area with massive accumulation of RER-derived vesicles with remnants of membrane-bound ribosomes. **(E,F)** Whorl **(E)** and parallel **(F)** arrays of RER cisterns in motor neurons from hSOD1^G93A^ mice. Note the presence of long segments with obliteration of the cisternal lumen and partial detachment of membrane-bound polyribosomes. Scale bars: **(A,B)** = 5 μm; **(C,D)** = 1 μm; **(E)** = 0.7 μm; **(F)** = 250 nm.

Although previous studies have demonstrated the induction of chromatolysis with fragmentation of the RER in motor neurons of both ALS patients and murine models of ALS (Kusaka et al., 1988; Oyanagi et al., 2008; Sasaki, 2010), we have investigated the cellular basis of RER alterations that leads to translational inhibition in the mutant SOD1 mice. Electron microscopy of motor neurons showed a dense cytoplasm with numerous typical stacks of RER cisterns in control neurons in contrast to the pale cytoplasm produced by the paucity of RER elements in chromatolytic transgenic neurons (Figures 2A,B). Detailed ultrastructural analysis of RER showed two characteristic alterations. The first consisted of focal dilatations of the RER cisterns with partial detachment of membrane-associated polyribosomes that ultimately leads to fragmentation of the cisterns in numerous small cytoplasmic vesicles ranging in diameter from 200 to 500 nm (Figures 2C,D). The RER origin of these vesicles was also supported by the presence of remnants of polyribosomes attached to the cytosolic face of the membrane (Figure 2D). The accumulation of RER-derived vesicles is a common finding in chromatolytic areas of transgenic hSOD1^G93A^ neurons. A second manifestation was the formation of whorl and stack arrays of RER containing collapsed cisterns with occlusion of the lumen (Figures 2E,F). Moreover, polyribosomes tended to be detached from collapsed cisterns, indicating that they are not engaged in active translations. This ultrastructural organization is distinct from the RER-derived lamellar bodies described in motor neurons of sporadic ALS patients (Sasaki, 2010). Taken together, these RER alterations reflect a severe disruption of the protein synthesis machinery.

### Compensatory response of the protein synthesis machinery to chromatolysis

**Figure 3:**
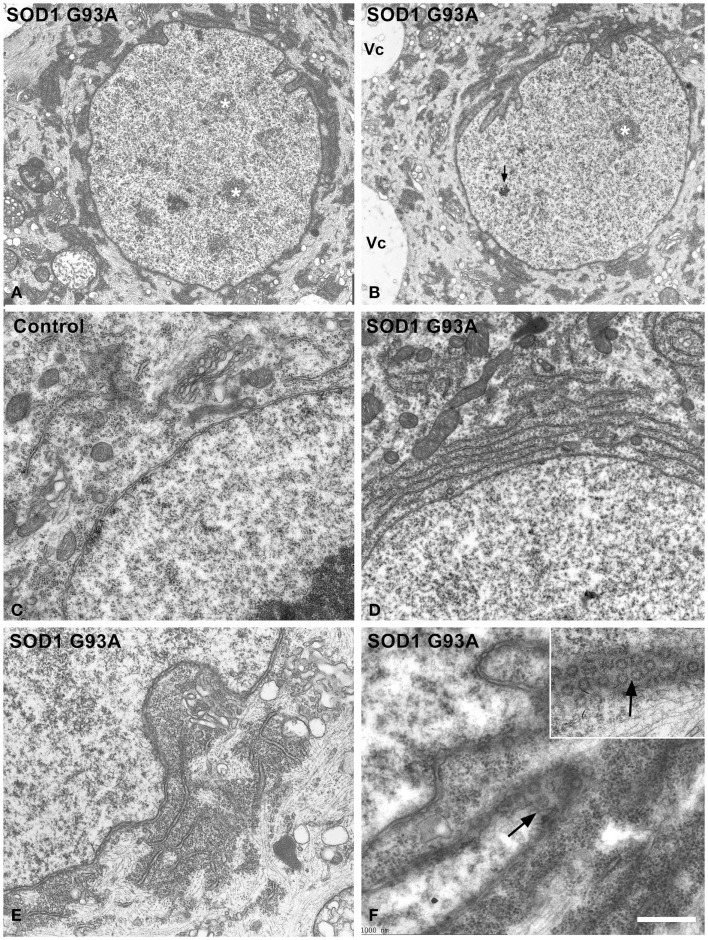
**(A–F)** Electron micrographs illustrating the organization of the perinuclear region in motor neurons from control **(C)** and hSOD1^G93A^ mice **(A,B,D–F)**. **(A,B)** Low magnification images of chromatolytic neurons showing the euchromatic nuclei with several interchromatin granule clusters (white asterisks) and infoldings of the nuclear envelope. The arrow indicates a Cajal body. Electron-dense cytoplasmic areas, corresponding to local accumulations of free polyribosomes and RER cisterns, appear concentrated at the perinuclear region. Note in **(B)**, a perinuclear cap of polyribosomes at the wrinkled nuclear pole and the presence of large cytoplasmic vacuoles (Vc). **(C–F)** Detail of the perinuclear region in control **(C)** and ALS **(D–F)** motor neurons. The perinuclear cytoplasm exhibits scattered polyribosomes, Golgi cisterns and some mitochondria in the control neuron **(C)**. In contrast, perinuclear accumulations of either concentric arrays of RER cisterns **(D)** or combinations of free polyribosomes and RER cisterns **(E)** are observed in an ALS motor neurons. Note in **(E,F)** that nuclear infoldings contain a great abundance of polyribosomes that fill the depressions of the nuclear envelope. Tangential sections of the nuclear membranes illustrate the great density of nuclear pores at the polyribosome-rich nuclear infoldings [arrows in **(F)** and inset]. Scale bars: **(A,B)** = 2.8 μm; **(C–E)** = 0.9 μm; **(F)** and inset = 600 nm.

**Figure 4:**
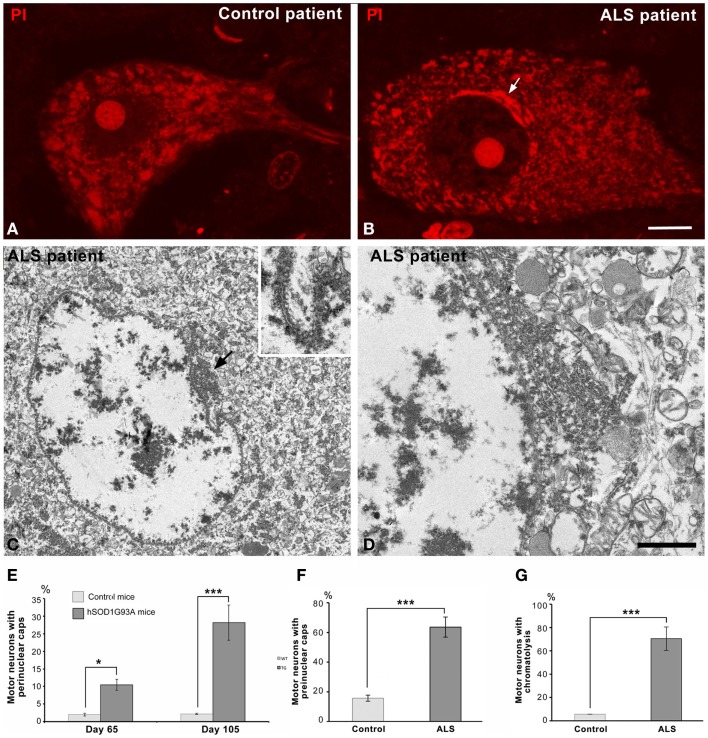
**(A,B)** Representative examples of human spinal motor neurons from the control **(A)** and the ALS patient **(B)** stained with propidium iodide. Note the typical organization of Nissl bodies in the control neurons and the perinuclear cap (arrow) in the chromatolytic ALS neuron. Both neurons exhibit a prominent nucleolus. Scale bar: **(A,B)**: 8 μm. **(C,D)** Electron micrographs of chromatolytic spinal motor neurons from a patient with sporadic ALS. Nuclei show a predominant euchromatin organization with some small aggregates of heterochromatin at the nuclear interior and associated with the nuclear envelope. Local accumulations of free polyribosomes and RER cisterns appear in the perinuclear cytoplasm, most often at nuclear infolding sites enriched with nuclear pores (arrow). Scale bar: **(C)** = 3 μm; **(D)** = 1 μm; inset: 0.85 μm. **(E)** Proportion of wild type and transgenic ALS motor neurons having perinuclear caps of RER. Data are mean ± SD; *p < 0.05, ***p < 0.001. **(F,G)** Quantitative analysis of the percentage of human control and ALS motor neurons with perinuclear caps **(F)** and chromatolysis **(G)**. At least 100 neurons from the control and the ALS patient were examined.

Next, we investigated possible cellular mechanisms involved in neuronal survival under conditions of severe chromatolysis in ALS motor neurons. In particular, we analyzed the reorganization of the RER. Both cytochemical staining with propidium iodide and toluidine blue staining showed the frequent presence of fluorescent or basophilic perinuclear caps enriched in RNA in motor neurons of the hSOD1^G93A^ mice at both presymptomatic and symptomatic stages (Figures 1B,C). The ultrastructural counterpart was the local accumulation of RER cisterns and free polyribosomes in close proximity to the nuclear envelope (Figures 3A,B). While in the majority of control neurons, the perinuclear cytoplasm commonly displayed scattered polyribosomes, Golgi complexes and mitochondria (Figure 3C), two main perinuclear arrangements of the protein synthesis machinery were found in the ALS mouse model. The first consisted of concentric arrays of RER cisterns in close proximity of the nuclear envelope (Figure 3D). The second were perinuclear caps of free polyribosome with some isolated cisterns of RER (Figure 3E). Perinuclear caps were most commonly observed in motor neurons with severe chromatolysis and abnormal accumulations of neurofilaments, and frequently occurred at a wrinkled nuclear pole in which the infoldings of the nuclear envelope were filled with polyribosomes and RER cisterns (Figures 3B,E). Tangential sections of the nuclear envelope at the nuclear infoldings showed high density of nuclear pores and their spatial association with polyribosomes (Figure 3F, inset). The quantitative analysis of the proportion of motor neurons having perinuclear caps of RER revealed a significant increase in ALS mice compared to wild type, with a notable higher frequency in symptomatic than in asymptomatic stages of the ALS (Figure 4E).

Interestingly, a similar reorganization of the protein synthesis machinery was observed in human motor neurons of the sporadic ALS patient. Figures 4A,B illustrate representative examples of a control motor neuron with typical Nissl bodies stained with propidium iodide, and an ALS patient motor neuron with chromatolysis and a strong fluorescent perinuclear cap. Electron microscopy analysis of chromatolytic neurons confirmed the perinuclear accumulations of RER cisterns and polyribosomes, which frequently appeared in nuclear infolding domains (Figures 4C,D). The quantitative analysis of the proportion of motor neurons with chromatolysis and/or perinuclear caps of RER showed a significant increase in the ALS patient as compared to the control patient (Figures 4F,G). Collectively, these findings suggest that the reorganization of the protein synthesis machinery at nuclear infoldings, which provide an increased nuclear surface studded with numerous nuclear pores, may facilitate nucleo-cytoplasmic traffic in chromatolytic neurons.

### Nucleolar configuration and transcriptional activity in motor neurons of the SOD1 mutant mice

**Figure 5:**
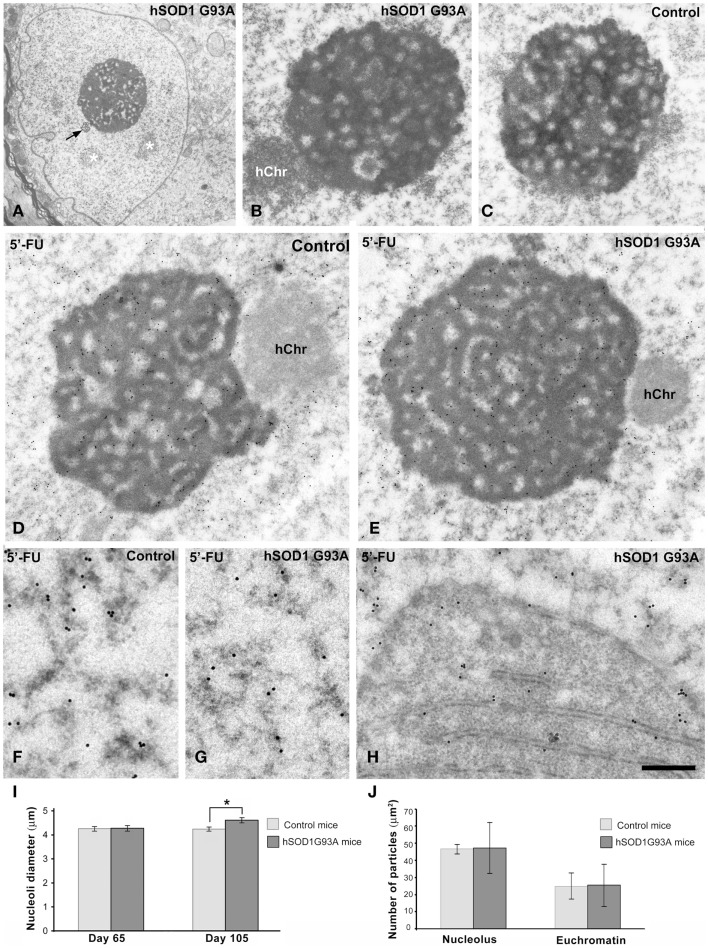
**(A–C)** Nucleolar organization in motor neurons from control **(C)** and ALS **(A,B)** hSOD1^G93A^ mice. **(A)** ALS chromatolytic neurons with nuclear eccentricity and a prominent nucleolus. Note the presence of interchromatin granule clusters (white asterisks) and a Cajal body (arrow). **(B,C)** In both control and ALS motor neurons, the nucleolus exhibits a typical reticulated configuration with numerous fibrillar centers surrounded by the dense fibrillar component and intercalated masses of the granular component. hChr: nucleolus-associated heterochromatin. **(D,E)** Transcription assay with a 45 min pulse of 5′-FU incorporation into nascent RNA. A similar pattern of 5′-FU incorporation is detected in nucleoli of both control and ALS motor neurons. Immunogold particles of 5′-FU incorporation in nucleolar transcription sites preferentially decorate the dense fibrillar component. Transcriptional activity is also detected throughout the euchromatin, while the transcriptionally silent heterochromatin (hChr) lacks of immunogold particles. **(F,G)** Detail of euchromatin regions from control and ALS motor neurons shows the localization of the extranucleolar transcription sites in perichromatin fibrils, which appear decorated with immunogold particles. (**H**) Some immunogold particles decorate newly synthesized RNA on polyribosomes accumulated at the perinuclear region, within a nuclear invagination, in a motor neuron from the hSOD1^G93A^ mouse. Scale bars: A = 2 μm; B,C = 0.8 μm; D,E = 0.75 μm; F–H = 325 nm. **(I)** Morphometric analysis of the nucleolar diameter in mononucleolated motor neurons from control and hSOD1^G93A^ mice. Data are mean ± SE from three independent experiments; *p < 0.05. At least 100 neurons per animal group were sampled. **(J)** Labeling density of gold particles detecting 5′-FU incorporation in euchromatin and nucleolar compartments. Data are mean ± SD (see additional information in Materials and Methods).

Since ribosome biogenesis is an essential step to sustain protein synthesis activity, we analyzed the response of nucleolus to severe chromatolysis in motor neurons of the ALS transgenic SOD1 mice. Light microscopy cytochemical staining with propidium iodide clearly illustrated prominent nucleoli in motor neurons from both control and ALS mice (Figure 1). The morphometric determination of the nucleolar diameter demonstrated that the nucleolar size was preserved in motor neurons from ALS mice (Figure 5I).

To determine whether the dysfunction of the protein synthesis caused by the severe chromatolysis modified the nucleolar architecture in motor neurons, we performed ultrastructural analysis. Both control and ALS mice nucleoli in motor neurons exhibited a typical reticulated configuration (Figures 5A–C) of neurons with high transcriptional activity (Peters et al., 1991; Casafont et al., 2006; Palanca et al., 2014b). This nucleolar organization is characterized by the presence of numerous fibrillar centers surrounded by a shell of dense fibrillar component and variable masses of granular component. The reticulated nucleolar architecture was well preserved even in motor neurons with nuclear eccentricity and severe chromatolysis of symptomatic hSOD1^G93A^ mice (Figures 5A). Moreover, nucleolar macrosegregation of dense fibrillar and granular components, an alteration of the neuronal nucleolus associated with the inhibition of the nucleolar transcription (Casafont et al., 2006, 2007; Baltanas et al., 2011a), was not observed.

Next, we investigated whether nucleolar transcription was affected in motor neurons of the hSOD1^G93A^ mice. We performed an *in situ* transcription assay at the ultrastructural level based on the incorporation of the RNA precursor 5′-FU into nascent RNA, following a 45 min pulse of intravenous administration of the halogenated nucleotide. Nucleolar and chromatin sites of 5′-FU incorporation were detected with the monoclonal anti-BrdU antibody and using a secondary antibody conjugated with gold particles. As illustrated in Figures 5D,E, a similar pattern of distribution of immunogold particles was observed in reticulated nucleoli of motor neurons from control and symptomatic ALS mice. Thus, immunogold particles preferentially decorated the threads of dense fibrillar component.

Another important point related to global transcriptional activity in motor neurons was the configuration of chromatin. Interestingly, ALS motor neurons with severe chromatolysis and vacuolar degeneration preserved the typical pale euchromatic nucleus of control neurons (Figure 1). This chromatin organization was confirmed by electron microscopy analysis in which only the interchromatin granule clusters, nuclear sites of accumulation of splicing factors, and post-transcriptional pre-mRNA processing (Melcák et al., 2000; Spector and Lamond, 2011), stand out on the predominant pattern of euchromatin (Figures 2A,B, 3A,B, and 5A). Furthermore, the 5′-FU transcription assay clearly demonstrated that the extranucleolar transcriptional activity was preserved in ALS motor neurons, as indicated the presence of numerous immunogold particles decorating perichromatin fibrils (Cmarko et al., 1999) throughout euchromatin domains (Figures 5F,G). As negative control, we illustrated the conspicuous absence of immunogold particles of 5′-FU incorporation in transcriptionally silent perinucleolar heterochromatin masses (Figures 5D,E). The quantitative analysis of the labeling density over the nucleolus and euchromatin, expressed as numbers of gold particles per 1 μm^2^, showed no significant differences in both transcription compartments between wild type and ALS motor neurons (Figure 5J). As expected, labeling density was higher in the nucleolus than in the euchromatin in both transgenic and wild type animals.

Finally, it is noteworthy that the presence of some immunogold particles decorating the polyribosomes that filled nuclear infoldings, indicating that this perinuclear domain of protein synthesis machinery contains newly synthesized RNAs exported from the nucleus (Figure 5H).

## Discussion

Neuropathological hallmarks in ALS spinal motor neurons include two main sequential stages: chromatolysis, with its associated vacuolar degeneration of perikaryal cytoplasm and neuronal processes, and apoptosis (Martin, 1999; Oyanagi et al., 2008; Sasaki, 2010). These neuronal alterations appear at the asymptomatic ALS and proceed faster during the symptomatic stage of the disease.

Our results in motor neurons of the hSOD1^G93A^ mouse and in the ALS patient indicate that the progression of chromatolysis associates with formation of SGs enriched in eIF3, a signature component of SGs required for their assembly (Ohn et al., 2008). Under physiological conditions translation initiation and translational silencing rates are in equilibrium and most cytoplasmic mRNA is located in polyribosomes (Anderson and Kedersha, 2008). Several studies indicate that ER stress shift this balance resulting in increased rate of translational silencing and sequestration of the excess of mRNAs released from polyribosomes in SGs (Kedersha et al., 2013). Recent biochemical studies have shown activation of ER stress pathways during motor neuron degeneration in the hSOD1^G93A^ mouse model (Kikuchi et al., 2006; Nagata et al., 2007; Saxena et al., 2009; Sasaki, 2010). Thus, the formation of SGs reported here in ALS motor neurons is consistent with an ER stress-induced chromatolytic disassembly of polyribosomes and subsequent recruitment of some released mRNAs into SGs, as being suggested by their cytochemical staining with propidium iodide. Moreover, previous studies in ALS cellular models and brain tissues from ALS patients have reported the recruitment of TDP-43 and FUS to SGs, two RNA-binding proteins involved in ALS pathogenesis (Volkening et al., 2009; Liu-Yesucevitz et al., 2010; Bentmann et al., 2012).

Previous electron microscopy studies of the RER in motor neurons of ALS patients have reported various types of alterations, such as cisternal distension with ribosomal detachment, intracisternal accumulations of amorphous material and, occasionally, formation of lamellar bodies, RER arrays with intercisternal electron-dense material (Oyanagi et al., 2008; Sasaki, 2010). Our ultrastructural analysis in motor neurons of the hSOD1^G93A^ mouse model suggest that, in addition to polyribosome detachment, the massive fragmentation of RER cisterns in numerous small vesicles with remnants of membrane-bound polyribosomes is a major structural component in chromatolytic areas of the cytoplasm. RER-derived small vesicles are clearly distinguishable from larger vacuoles of several micrometers in diameter characteristic of the vacuolar degeneration in ALS motor neurons (Sasaki et al., 2004). Another alteration frequently found in RER arrays is the occlusion of the cisternal lumen accompanied by polyribosome detachment. This structural modification can prevent protein synthesis and peptide translocation and processing in the RER lumen. Taken together, these RER alterations provide new structural basis for the disruption of Nissl bodies and translational arrest in chromatolytic neurons.

However, it is worth noting that in some experimental models of axotomy and neurotoxicity chromatolysis is a reversible dysfunction of protein synthesis machinery, indicating the activation of neuroprotective mechanisms leading to neuronal recovery of function (Kinderman and Jones, 1993; Palanca et al., 2014a). In the current hSOD1^G93A^ mouse model, we show that chromatolysis induces compensatory neuronal mechanisms, which might allow chromatolytic motor neurons tolerate a severe dysfunction of proteostasis until the final activation of apoptosis. Such mechanisms include the perinuclear reorganization of the protein synthesis machinery, the higher-order chromatin organization in predominant active euchromatin, and the nucleolar activity in ribosome biogenesis.

An important observation in motor neurons from both hSOD1^G93A^ mice and the ALS patient is the preferential perinuclear reorganization of protein synthesis machinery, in close proximity to the nuclear envelope. It is well known that the nuclear envelope environment provides a specialized region for a wide range of cellular functions, such as signal transduction from cytoskeleton to nucleus, regulation of nuclear morphology, transcription at the nuclear periphery and nucleo-cytoplasmic traffic (for a review, see Mekhail and Moazed, 2010; Wilczynski, 2014). A similar perinuclear reorganization of RER and free polyribosomes has been reported in recovery neurons from axotomy or proteasome inhibition (for a review, see Lieberman, 1971; Palanca et al., 2014a). This perinuclear organization is particularly prominent at the dendritic nuclear pole of normal Purkinje cells (Palay and Chan-Palay, 1974), where the local accumulation of the protein synthesis machinery seems to facilitate the transfer of new-synthesized proteins and mRNAs to the dendritic tree. In the case of ALS, motor neuron accumulation of RER and free polyribosomes frequently occur at sites of nuclear infoldings, where increased nuclear surface is studded with high density of nuclear pores. This spatial arrangement decreases diffusion distances of RNAs from the nucleus to protein synthesis machinery, likely facilitating perinuclear translation at the proximity of nuclear pores. In fact, we have observed that newly synthesized RNAs are rapidly exported to the protein synthesis machinery that filled nuclear infoldings after a short pulse of 45 min of 5′-FU administration. Moreover, in an animal model of hippocampal neurons stimulation, the induction of nuclear infoldings has also been related to enhancing transcription at the proximity of nuclear pores (Akhtar and Gasser, 2007; Wittmann et al., 2009). In this context, we consider that the perinuclear reorganization of protein synthesis machinery reflects a compensatory reactive response to chromatolysis, in order to preserve the translational activity required for neuronal survival. The molecular mechanisms underlying ER biogenesis at the perinuclear region in response to the disturbance of ER proteostasis are unknown. The ER is a dynamic compartment that can be expanded according to cellular demands of secretory proteins, although the molecular mechanisms that regulate the synthesis of phospholipid and proteins required for its biogenesis are poorly understood (Sriburi et al., 2004). In the context of ER stress in neurodegenerative disorders, the adaptive outcome of the UPR includes the activation of the spliced form of the transcription factor XBP1s (X-box binding protein) that has neuroprotective activity (Casas-Tinto et al., 2011; Hetz and Mollereau, 2014). Interestingly, cellular and biochemical studies have demonstrated that this factor links mammalian UPR to phospholipid biosynthesis and ER biogenesis (Sriburi et al., 2004). Further studies will be needed to determine whether XBP1s is involved in the perinuclear reorganization of ER observed in ALS motor neurons.

Regarding chromatin organization in ALS motor neurons, what is noteworthy is their predominant euchromatin pattern, a chromatin architecture associated with permissive transcriptional activity (for a review, see Wilczynski, 2014). Indeed, the 5′-FU incorporation assay clearly demonstrated that the transcriptional activity is preserved in the extensive euchromatin domains where immunogold particles decorate perichromatin fibrils, nuclear sites of transcription, and cotranscriptional pre-mRNA processing enriched in nascent pre-mRNA transcripts (Cmarko et al., 1999; Casafont et al., 2006; Spector and Lamond, 2011). Maintenance of euchromatin architecture and transcriptional activity in ALS motor neurons could be essential for the activation of genes involved in the neuronal stress response to the disturbance of ER proteostasis (Steffen et al., 2010; Hetz and Mollereau, 2014) and consequently for neuronal survival. In contrast, in the *pcd* (“Purkinje cell degeneration”) mouse model of neurodegeneration, large-scale chromatin condensation is associated with transcriptional silencing, absence of cellular signs of neuroprotection, and rapid induction of apoptosis in Purkinje cells (Baltanas et al., 2011b).

Concerning the nucleolar response to chromatolysis in motor neurons of the hSOD1^G93A^ mice, it is noteworthy that the preservation of the size, reticulated configuration, and high transcriptional activity of the nucleolus under conditions of severe chromatolysis. Similarly, a high rate of rRNA synthesis has been reported in experimental models of neuronal injury with severe chromatolysis, such as axotomy and treatment with proteasome inhibitors, which trigger a neuroprotective compensatory response to promote functional recovery (Kinderman and Jones, 1993; Wells and Vaidya, 1994; Palanca et al., 2014b). Interestingly, motor neurons in the ALS mouse model preserve a reticulated nucleolar configuration with numerous fibrillar centers, a reliable feature of transcriptionally very active neurons (Lafarga et al., 1991; Peters et al., 1991; Pena et al., 2001; Berciano et al., 2007), in addition to a high nucleolar incorporation of 5′-FU into nascent rRNAs. These results support an active participation of nucleolar transcription as compensatory mechanisms to chromatolysis. These findings are also consistent with our recent study (Palanca et al., 2014b) demonstrating high nucleolar incorporation of 5′-FU and upregulation of genes encoding UBF, fibrillarin, and B23, three essential proteins for rRNA synthesis and processing (Boisvert et al., 2007; Hernandez-Verdun et al., 2010; Grummt, 2013), as a neuroprotective compensatory response to proteasome inhibition. In contrast, in Parkinson’s disease and other neurodegenerative disorders with severe nucleolar dysfunction, the impairment of rRNA transcription and disruption of nucleolar integrity cause nucleolar stress (Baltanas et al., 2011a; Hetman and Pietrzak, 2012; Parlato and Kreiner, 2013; Parlato and Liss, 2014).

In this scenario, the high rRNA transcription observed in ALS motor neurons of the hSOD1^G93A^ mice could be a compensatory attempt by chromatolytic neurons to enhance ribosome biogenesis required for neuronal survival. Epigenetic activation of chromatin and nucleolar transcription could have important therapeutic implications for neuroprotection in ALS and other neurodegenerative disorders. Although histone deacetylase inhibitors are currently used as therapeutic agents, we raise the untapped potential of the nucleolar transcription of ribosomal genes as an exciting new target for the therapy of some neurodegenerative diseases.

## Conflict of Interest Statement

The authors declare that the research was conducted in the absence of any commercial or financial relationships that could be construed as a potential conflict of interest.
